# Lung Cancer Screening Among U.S. Military Veterans by Health Status and Race and Ethnicity, 2017–2020: A Cross-Sectional Population-Based Study

**DOI:** 10.1016/j.focus.2023.100084

**Published:** 2023-02-09

**Authors:** Alison S. Rustagi, Amy L. Byers, James K. Brown, Natalie Purcell, Christopher G. Slatore, Salomeh Keyhani

**Affiliations:** 1 Division of General Internal Medicine, Medical Service, San Francisco Veterans Affairs Health Care System, San Francisco, California; 2 Department of Medicine, University of California, San Francisco, California; 3 Research Service, San Francisco Veterans Affairs Health Care System, San Francisco, California; 4 Department of Psychiatry and Behavioral Sciences, University of California, San Francisco, California; 5 Weill Institute for Neurosciences, University of California, San Francisco, California; 6 Division of Pulmonary, Critical Care, and Sleep Medicine, Medical Service, San Francisco Veterans Affairs Health Care System, San Francisco, California;; 7 Integrative Health, San Francisco Veterans Affairs Health Care System, San Francisco, California;; 8 Social Behavioral Health Sciences, School of Nursing, University of California, San Francisco, California; 9 National Center for Lung Cancer Screening, Veterans Health Administration, Washington, District of Columbia; 10 Division of Pulmonary and Critical Care Medicine, VA Portland Health Care System, Portland, Oregon; 11 Division of Pulmonary & Critical Care Medicine, Department of Medicine, Oregon Health & Science University School of Medicine, Portland, Oregon

**Keywords:** Lung cancer screening, lung cancer, veterans, race, ethnicity, health disparities

## Abstract

**Introduction:**

Veterans are at high risk for lung cancer and are an important group for lung cancer screening. Previous research suggests that lung cancer screening may not be reaching healthier and/or non-White individuals, who stand to benefit most from lung cancer screening. We sought to test whether lung cancer screening is associated with poor health and/or race and ethnicity among veterans.

**Methods:**

This cross-sectional, population-based study included veterans eligible for lung cancer screening (aged 55–79 years, ≥30 pack-year smoking history, current smokers or quit within 15 years, no previous lung cancer) in the 2017–2020 Behavioral Risk Factor Surveillance System surveys. Exposures were (1) *poor health*, defined as fair/poor health status and difficulty walking or climbing stairs, aligning with eligibility criteria for a pivotal lung cancer screening trial, and (2) race/ethnicity. The outcome was a receipt of lung cancer screening. All variables were self-reported.

**Results:**

Of 3,376 lung cancer screening–eligible veterans representing an underlying population of 866,000 individuals, 20.3% (95% CI=17.3, 23.6) had poor health, and 13.7% (95% CI=10.6, 17.5) identified as non-White. Poor health was strongly associated with lung cancer screening (adjusted RR=1.64, 95% CI=1.06, 2.27); one third of veterans screened for lung cancer would not qualify for a pivotal lung cancer screening trial in terms of health. Marked racial disparities were observed among veterans: after adjustment, non-White veterans were 67% less likely to report lung cancer screening than White veterans (adjusted RR=0.33, 95% CI=0.11, 0.66).

**Conclusions:**

Lung cancer screening is correlated with poorer health and White race/ethnicity among veterans, which may undermine its population-level effectiveness. These results highlight the need to promote lung cancer screening, especially for healthier and/or non-White veterans, an important group of Americans for lung cancer screening.

## Highlights

• Among veterans, poorer health is tied to the use of lung cancer screening.
• One third of veterans screened for lung cancer would not meet the health requirements of a pivotal lung cancer screening trial.
• Marked racial and ethnic disparities exist in lung cancer screening among veterans.
• Non-White veterans are 67% less likely to be screened than White veterans.

## INTRODUCTION

Lung cancer is the deadliest cancer in the U.S.^1^ Screening through low-dose computerized tomography (LDCT) can reduce death from lung cancer by 16%–24%^2^, ^3^, ^4^ among those without substantial comorbidities. Veterans are likely to benefit substantially from LCS: veterans have higher smoking prevalence^5^ and are of older age^6^ than nonveterans, which may increase lung cancer risk, and unique occupational exposures^7^^,^^8^ may further increase veterans’ cancer risk.^9^ Veterans are an important population to consider for lung cancer screening (LCS).

However, the general population of veterans also has poorer health than nonveterans,^6^ which can preclude surgery to cure early lung cancer^10^ and increase the risk of dying from competing causes of death. Thus, poor health could attenuate the benefit of early lung cancer detection^10^, ^11^, ^12^, ^13^ and expose veterans to unjustified risk of immediate harm.^14^ The 2 trials that showed the mortality benefit of LCS—the Nederlands-Leuvens Longkanker Screenings Onderzoek (NELSON) trial^4^ in Europe and the National Lung Screening Trial (NLST) in the U.S.—were restricted to those healthy enough to be candidates for thoracic surgery, for 2 reasons: (1) the mortality benefit of LCS is thought to be driven by the surgical cure of early disease^10^^,^^15^ and (2) competing causes of death could overwhelm any benefit of early lung cancer detection.^11^ The value of LCS for those in poor health is unproven. Furthermore, modeling indicates that screening those with <5-year life expectancy results in more harm with no additional benefits at a population level.^13^

To quantify health, the NELSON trial excluded those who rated their health as moderate or bad and who had difficulty climbing stairs,^4^ making self-rated health and ability to climb stairs valuable points of comparison in real-world data. The National Lung Screening Trial (NLST) used an individualized clinical assessment that cannot be replicated as easily^3^; on average, NLST participants had 1.2 comorbidities.^16^

In addition to health status, race and ethnicity are important to consider: LCS provided a greater reduction in lung cancer–specific and all-cause mortality for Black participants in the NLST.^16^ It is unknown whether overall LCS use differs by race and ethnicity among veterans. Previous analyses in the Veterans Health Administration (VHA),^17^ which serves approximately 49% of veterans,^18^^,^^19^ have shown increased acceptance of screening among Black and Hispanic veterans after being offered LCS but also increased risk of delayed or no follow-up after initial LDCT scans among Black veterans.^20^

If LCS is used more frequently among sicker individuals and/or racial disparities exist in its use, the population-level mortality benefit of LCS in the real world may be less than that seen in randomized trials. Previous work by our group using population-based survey data has shown that LCS is higher among those in poorer health and among those of non-Hispanic White race and ethnicity.^21^ It is unknown whether these patterns of LCS use hold true for U.S. military veterans, a unique and important subgroup to consider for LCS.

Understanding the current use of LCS in veterans would provide useful public health information to maximize veterans’ benefits and be a benchmark for ongoing implementation of LCS given this group's uniquely elevated risk of lung cancer. This study sought to test our hypothesis that the use of LCS among U.S. military veterans differed by (1) *health status*, defined using criteria similar to those of the pivotal NELSON trial, and/or (2) race and ethnicity, with those in better overall health and of non-White race/ethnicity less likely to receive LCS.

## METHODS

### Study Population

This cross-sectional, population-based study utilized data from the 2017–2020 data sets of the Behavioral Risk Factor Surveillance System (BRFSS), an annual phone survey of community-dwelling U.S. adults conducted by the Centers for Disease Control and Prevention.^22^ BRFSS conducts >400,000 interviews with residents of all 50 states, the District of Columbia, and 3 territories to ascertain behaviors related to health and health service utilization. Core questions are asked of all respondents; states may opt to add additional topic-specific modules. LCS eligibility and use are included in an optional module that was conducted in a subset of states, representing 28 unique states in total across the 2017–2020 survey years.^23^, ^24^, ^25^, ^26^ We identified U.S. veterans eligible for LCS by age and smoking history on the basis of 2013 U.S. Preventive Services Task Force guidelines and limited the sample to individuals aged 55–79 years with a ≥30 pack-year smoking history and current smokers or former smokers who quit within 15 years before. Because BRFSS aggregates ages above 79 years into 1 category (80+ years), we could not isolate individuals who were aged 80 years from those who were above the age cut-off for LCS; thus, we excluded those aged 80+ years from the analysis. Veteran status was ascertained with the question (yes/no): *Have you ever served on active duty with the U.S. Armed Forces, either in the regular military or in a National Guard or reserve military unit?* Smoking behavior was assessed with the questions, *How old were you when you first started to smoke cigarettes regularly?, How old were you when you last smoked cigarettes regularly?*, and *On average, when you [smoke/smoked] regularly, about how many cigarettes [do/did] you usually smoke each day?* Exclusion criteria were personal history of lung cancer and being aged <55 or ≥80 years. Individuals with missing values for key eligibility variables were excluded from the analysis (Appendix Figure, available online, shows the study flowchart).

This study utilized publicly available, deidentified data and was exempt from IRB review. Reporting conformed to the STROBE reporting guideline for cross-sectional studies.^27^

### Measures

The primary exposures were health status and race and ethnicity. Health status was categorized into a binary variable indicating whether a respondent met/did not meet criteria similar to the initial eligibility criteria of the NELSON trial; the BRFSS questionnaire wording differed slightly from that of the NELSON trial screening questionnaire. NELSON trial investigators excluded those who rated their health as moderate or bad and who could not climb 2 flights of stairs. For this analysis, health status was ascertained using 2 survey questions: (1) self-rated health: *Would you say that in general your health is – Excellent, Very Good, Good, Fair, Poor?* and (2) ability to climb stairs (yes/no): *Do you have serious difficulty walking or climbing stairs?* Thus, individuals who rated their health as fair or poor and reported serious difficulty in walking or climbing stairs would not meet trial eligibility criteria and were categorized as having poor health; those who rated their health as excellent, very good, or good and/or reported no difficulty in walking or climbing stairs likely met initial trial eligibility criteria and were categorized as having good health. Health status was missing for *n*=8 veterans in the final sample (0.2%).

Race/ethnicity was self-reported with the following categories defined by the BRFSS survey: White non-Hispanic, Black or African American non-Hispanic, American Indian or Alaska Native non-Hispanic, Asian non-Hispanic, Native Hawaiian or other Pacific Islander non-Hispanic, other race non-Hispanic, multiracial non-Hispanic, and Hispanic. In the main analysis, race and ethnicity were included in the models as a 2-category variable (non-Hispanic White [White] or not non-Hispanic White [non-White]) for model stability because the unweighted numbers of screened individuals in racial/ethnic subgroups other than White were <30. To confirm with reporting standards,^28^ analyses disaggregated by race and ethnicity subgroups were also conducted. Race and ethnicity were missing for *n*=66 veterans in the final sample (2.0%).

The primary outcome was self-reported LCS, ascertained in the optional Lung Cancer Screening module with the question, *In the last 12 months, did you have a CT or CAT scan?* A respondent was counted as screened if they chose *Yes, to check for lung cancer.* Screening status was missing for *n*=59 veterans who would otherwise have been included in the final sample (1.7% of otherwise eligible individuals).

We chose covariates a priori on the basis of previous research and our own previous analyses.^21^ Broadly, covariates included demographic factors,^29^ socioeconomic factors and smoking behavior,^29^^,^^30^ healthcare coverage and access,^31^ chronic obstructive pulmonary disease (COPD), previous cancer history,^32^, ^33^, ^34^ and receipt of previous vaccination^29^ as a marker of preventive health service utilization.

Variables on SES and demographics included age, sex, marital status, and educational attainment. To describe health factors and healthcare access, we captured smoking pack-year history, BMI (kg/m^2^), health insurance status, receipt of influenza vaccine in the previous 12 months, and difficulty in paying for medical care, which was ascertained with the question: *Was there a time in the past 12 months when you needed to see a doctor but could not because of cost?* Diagnosis of COPD was assessed with the question, *Were you ever told you had chronic obstructive pulmonary disease, COPD, emphysema, or chronic bronchitis?* Personal history of nonskin, nonlung cancer was assessed with 2 questions: [*Has a doctor, nurse, or other health professional ever told you that you had any of the following…. other [non-skin] types of cancer?* and *What type of cancer was it? Any functional limitation* was defined as a self-report of any of the following: difficulty in walking or climbing stairs, dressing or bathing, or running errands. Number of comorbidities (0, 1, 2, or ≥3) was the sum of the following self-reported comorbidities: chronic kidney disease, arthritis, COPD, asthma, vascular disease, diabetes, and personal history of cancer other than skin cancer.

### Statistical Analysis

We conducted univariate analyses for variables of interest, calculating *p*-values from chi-square tests to evaluate differences in LCS prevalence. To account for the complex survey design and produce population-representative estimates, we implemented in analyses survey weights, clustering, and stratification following standardized protocols from the Centers for Disease Control and Prevention, with standard errors calculated using Taylor-linearized variance estimation.^35^ Chi-square tests used the Rao–Scott correction F-statistic to account for complex survey design.^36^ Design-adjusted estimates and tests were not computed when a table cell had zero frequency. We used multivariable logistic regression to examine the association between LCS and health status and LCS and race and ethnicity, adjusted for sociodemographic and health factors. Because the baseline prevalence of LCS was >10% in the nonexposed groups, we then converted ORs to RRs using the formula by Zhang and Yu.^37^

For covariates with >2 categories, each category was included as a dummy variable in regression models to maximize model flexibility. All tests were 2 sided values of *p*<0.05 were considered significant.

Analyses were conducted in Stata (Version 17.0, College Station, TX) between August 2021 and June 2022. Individuals with missing data were excluded from the analysis. Given the low prevalence of missing data (below the accepted threshold of 5%), a complete case analysis would if anything be conservative and tend to bias our estimates toward the null.^38^

## RESULTS

Across 2017–2020, 332,076 individuals responded to the survey, and 138,955 respondents were aged 55–79 years without a personal history of lung cancer and had complete smoking data and known veteran status, representing an underlying population of 33.7 million individuals (Appendix Figure, available online, shows the study flowchart). Of these, 14,536 were eligible for LCS with known screening status; of these, 3,376 identified as military veterans, representing a population of 866,000 veterans. Veterans were much more likely to be eligible for LCS (17.0%, 95% CI=15.7–18.2) than nonveterans (10.2%, 95% CI=9.7–10.7; *p*<0.001) (Appendix Table 1, available online). Veterans were overrepresented among LCS-eligible individuals: veterans made up 14.7% (95% CI=14.2, 15.3) of those aged 55–79 years regardless of smoking history versus 23.2% (95% CI=21.5, 24.9) of those aged 55–79 years who were eligible for LCS owing to heavy smoking history. Self-reported LCS was more common among veterans (19.4%, 95% CI=15.8, 22.9) than among nonveterans (16.3%, 95% CI=14.0, 18.5), although not to a significant degree.

The majority (60.6%, 95% CI=56.6, 64.4) of LCS-eligible veterans were aged ≥65 years, 94.3% (95% CI=92.2, 95.9) were male, and nearly half (46.1%, 95% CI=42.2, 50.1) had a ≥50 pack-year history of tobacco use (Table 1). Most veterans (79.7%, 95% CI=76.4, 82.7) would have met the basic eligibility criteria for the NELSON trial in terms of health status (i.e., endorsing excellent/very good/good overall health and/or no difficulty in walking or climbing stairs). The majority (86.3%, 95% CI=82.5, 89.4) of veterans identified as non-Hispanic White; the next largest group defined by self-reported race and ethnicity was non-Hispanic Black (9.1%, 95% CI=6.5, 12.6) (Appendix Table 2, available online).

**Table 1:** Demographic and Health Factors of Veterans Eligible for Lung Cancer Screening in 28 States, 2017–2020

Demographic or health factor	Veterans (unweighted *n*=3,376), weighted % (95% CI)
Health status^a^	
Good health	55.6 (51.7, 59.5)
Poor health	44.4 (40.5, 48.3)
Self-rated general health^b^	
Excellent	6.0 (4.5, 8.1)
Very good	21.9 (9.0, 25.1)
Good	38.5 (34.6, 42.5)
Fair	22.4 (19.2, 25.9)
Poor	11.2 (9.0, 13.9)
Difficulty walking or climbing stairs^c^	
No	69.3 (65.6, 72.8)
Yes	30.7 (27.2, 34.4)
Race and ethnicity^d^	
White	86.3 (82.5, 89.4)
Non-White	13.7 (10.6, 17.5)
Age^e^	
50–64 years	39.4 (35.6, 43.4)
65–79 years	60.6 (56.6, 64.4)
Sex^e^	
Male	94.3 (92.2, 95.9)
Female	5.7 (4.1, 7.8)
Personal doctor^f^	
Yes	86.4 (83.8, 88.7)
No	13.6 (11.3, 16.2)
Difficulty dressing or bathing^g^	
No	90.6 (88.2, 92.6)
Yes	9.4 (7.4, 11.8)
Difficulty doing errands alone^h^	
No	90.0 (87.6, 92.0)
Yes	10.0 (8.0, 12.4)
Any functional limitation^i^	
No	67.4 (63.6, 71.0)
Yes	32.6 (29.0, 36.4)
Smoking history, in pack-years^e^^,^^j^	
30–<40	24.0 (20.4, 28.0)
40–<50	29.9 (26.4, 33.6)
≥50	46.1 (42.2, 50.1)
Comorbidities, total number^e^^,^^k^	
0	25.6 (22.0, 29.5)
1	30.2 (26.7, 33.9)
2	21.5 (18.7, 24.6)
≥3	22.7 (19.5, 26.3)
Chronic kidney disease^l^	
No	93.8 (91.9, 95.4)
Yes	6.2 (4.6, 8.1)
Arthritis^m^	
No	51.4 (47.4, 55.4)
Yes	48.6 (44.6, 52.6)
Chronic obstructive pulmonary disease^n^	
No	67.5 (63.7, 71.1)
Yes	32.5 (28.9, 36.3)
Asthma^o^	
No	91.9 (89.3, 93.8)
Yes	8.1 (6.2, 10.7)
Vascular disease^p^	
No	68.8 (65.1, 72.2)
Yes	31.2 (27.8, 34.9)
Diabetes^q^	
No	79.2 (76.2, 81.9)
Yes	20.8 (18.1, 23.8)
Personal history of cancer^g^	
No	85.5 (82.7, 87.8)
Yes	14.5 (12.2, 17.3)

a *Health status* was defined to align with NELSON trial eligibility criteria. Specifically, *good health* was equivalent to *potentially eligible for NELSON trial participation*, defined as self-rated health excellent, very good or good, and no difficulty walking up stairs. *Poor health* was equivalent to *ineligible for NELSON trial participation*, defined as fair or poor health and/or difficulty in walking up stairs. Missing for *n*=10.

b Missing for *n*=11.

c Missing for *n*=12.

d Race and ethnicity were included in the models as a 2-category variable (non-Hispanic White [White] or not non-Hispanic White [non-White]) for model stability because the unweighted numbers of screened individuals in racial/ethnic subgroups other than non-Hispanic White were <30. The non-White group included the following: Black or African American non-Hispanic, American Indian or Alaska Native non-Hispanic, Asian non-Hispanic, Native Hawaiian or other Pacific Islander non-Hispanic, other race non-Hispanic, multiracial non-Hispanic, and Hispanic. Missing for *n*=66.

e No missing data.

f Missing for *n*=17.

g Missing for *n*=4.

h Missing for *n*=8.

i Self-report of any of the following: difficulty in walking or climbing stairs, dressing, bathing, or running errands. Missing for *n*=12.

j Average number of packs smoked per day, multiplied by the duration of smoking in years.

k Sum of the following self-reported comorbidities: chronic kidney disease, arthritis, chronic obstructive pulmonary disease, asthma, vascular disease, diabetes, and personal history of cancer other than skin cancer.

l Missing for *n*=15.

m Missing for *n*=28.

n Missing for *n*=14.

o Missing for *n*=30.

p Previous myocardial infarction, coronary heart disease, and cerebrovascular accident. Missing for *n*=5.

q Missing for *n*=2.

g Excluding skin cancer. Individuals with a personal history of lung cancer were excluded from the analysis. Missing for *n*=19. NELSON, Nederlands-Leuvens Longkanker Screenings Onderzoek.

Overall, 32.6% (22.8%–44.2%) of veterans screened for lung cancer would not have qualified for the NELSON trial of LCS that showed its efficacy. Veterans who were not healthy enough to meet initial NELSON eligibility criteria were significantly more likely to report LCS than healthier veterans: of NELSON-ineligible veterans, 31.2 (20.9–41.4) reported LCS compared with just 16.4 (13.1–19.7) of those who were eligible (*p*=0.002) (Table 2). After adjustment for sociodemographic and health characteristics and race and ethnicity, veterans who did not meet initial eligibility criteria for NELSON owing to poor health were 1.64 times more likely to receive screening than those who did meet NELSON eligibility (adjusted RR=1.64, 95% CI=1.06, 2.27).

**Table 2:** Use of LCS Among Veterans With/Without Poor Health Defined Using NELSON Trial Eligibility Criteria, 2017–2020

Health status^a^	Total LCS-eligible (unweighted *n*)	Total received LCS (unweighted *n*)	Proportion received LCS (weighted proportion, 95% CI)	Unadjusted RR (95% CI)	Adjusted RR (95% CI)^b^
Good health	2,244	415	16.4 (13.1, 19.7)	1.00 (ref)	1.00 (ref)
Poor health	553	156	31.2 (20.9, 41.4)	**1.90 (1.29, 2.50)**	**1.64 (1.06, 2.27)**

*Note:* Boldface indicates statistical significance (*p*<0.05).

a *Health status* was defined to align with NELSON trial eligibility criteria. Specifically, *good health* was equivalent to *potentially eligible for NELSON trial participation*, defined as self-rated health excellent, very good or good, and no difficulty in walking up stairs. *Poor health* was equivalent to *ineligible for NELSON trial participation*, defined as fair or poor health and/or difficulty in walking up stairs.

b Adjusted for race (White/non-White), age (5-year categories), sex (male/female), marital status (married/divorced/widowed/separated/never married/part of an unmarried couple), BMI (<18.5 kg/m^2^, 18.5–<25 kg/m^2^, 25–<30 kg/m^2^, 30+ kg/m^2^), education (never attended school or only kindergarten/elementary or middle school/some high school/high school graduate/some college/college graduate or more), smoking history in pack-years (quartiles), health insurance status (any/none), receipt of influenza vaccine in previous 12 months (no/yes), difficulty in paying for medical care (no/yes), personal history of nonlung cancer (no/yes), and survey year (2017/2018/2019/2020). LCS, lung cancer screening; NELSON, Nederlands-Leuvens Longkanker Screenings Onderzoek.

The use of LCS differed significantly by self-identified race and ethnicity among veterans: only 6.2% (2.5%–10.0%) of non-White veterans reported LCS, compared with 21.7% (17.7%–25.7%) of White veterans (*p*<0.001) (Table 3). After adjusting for sociodemographic and health covariates, including health status, significant racial and ethnic disparities in LCS use persisted among veterans: non-White veterans were 67% less likely to report LCS than White veterans (adjusted RR=0.33, 95% CI=0.16, 0.66). Analyses disaggregated by self-reported race and ethnicity are presented in Appendix Tables 2 and 3 (available online); owing to model instability from small absolute numbers of screened individuals, these point estimates should be interpreted with caution.

**Table 3:** Use of LCS Among Veterans by Race and Ethnicity, 2017–2020

Race and ethnicity^a^	Total LCS-eligible (unweighted *n*)	Total received LCS (unweighted *n*)	Proportion received LCS (weighted proportion, 95% CI)	Unadjusted RR (95% CI)	Adjusted RR (95% CI)^b^
White	2,987	521	21.7 (17.7, 25.7)	1.00 (ref)	1.00 (ref)
Non-White	328	41	6.2 (2.5, 10.0)	**0.29 (0.14, 0.56)**	**0.33 (0.16, 0.66)**

*Note:* Boldface indicates statistical significance (*p*<0.05).

a Race and ethnicity were included in the models as a 2-category variable (non-Hispanic White [White] or not non-Hispanic White [non-White]) for model stability because the unweighted numbers of screened individuals in racial/ethnic subgroups other than non-Hispanic White were <30.

b Adjusted for health status as measured by NELSON eligibility (good health/poor health), age (5-year categories), sex (male/female), marital status (married/divorced/widowed/separated/never married), BMI (<18.5 kg/m^2^, 18.5–<25 kg/m^2^, 25–<30 kg/m^2^, 30+ kg/m^2^), education (never attended school or only kindergarten/elementary/middle school/some high school/high school graduate/some college/college graduate or more), smoking history in pack-years (quartiles), health insurance status (any/none), receipt of influenza vaccine in previous 12 months (no/yes), difficulty in paying for medical care (no/yes), diagnosis of chronic obstructive pulmonary disease (no/yes), personal history of nonlung cancer (no/yes), and survey year (2017/2018/2019/2020). LCS, lung cancer screening; NELSON, Nederlands-Leuvens Longkanker Screenings Onderzoek.

## DISCUSSION

In this population-based, cross-sectional analysis of 3,376 respondents representing 866,000 veterans, veterans represented nearly one quarter of all LCS-eligible Americans. One third (33%) of LCS among veterans reached those who would not qualify for the pivotal NELSON trial on the basis of poor health. Furthermore, veterans with poor health were 64% more likely to report screening than healthier veterans. Marked racial and ethnic disparities in LCS use were present in veterans, with non-White veterans 67% less likely to receive screening than White veterans.

Our previous research indicates that LCS is more common among less healthy individuals, using a variety of measures of health status.^21^ This study suggests that the association between poor health and LCS use holds true for veterans. We defined *poor health* as rating one's health as fair or poor and endorsing significant limitations with walking or climbing stairs, in line with the NELSON trial exclusion criteria, 1 of 2 randomized trials that showed the mortality benefit of LCS. These patterns of LCS use may limit the benefit of LCS owing to competing causes of death and/or inability to receive curative lung surgery for early-stage disease.^10^^,^^15^ Randomized trials that showed the mortality benefit of LCS (NLST, NELSON) were restricted to healthy individuals without significant functional limitations^3^^,^^4^; the benefit of screening outside this group is unknown.^10^

For all screening tests, patients and their clinicians must weigh the immediate risks of screening against the possible long-term benefit; screening is only justified if an individual is healthy enough to likely live to see the benefits of screening.^14^ For LCS specifically, it is unclear how to identify those who are healthy enough to justify the short-term risks of LCS, which include radiation, invasive diagnostic procedures and their complications, false positive results, overdiagnosis, anxiety, and others.^13^ Weighing immediate risks against long-term benefits may be particularly important for veterans: among veterans screened in the VHA, those who needed invasive procedure(s) for an abnormal screening result experienced approximately 2 times more procedural complications than participants in the NLST, with 20% of veterans experiencing a major/intermediate complication within 10 days of invasive procedure.^39^

National guidelines use a variety of health metrics to define being healthy enough for LCS. The U.S. Preventive Services Task Force recommends LCS for those willing and able to undergo thoracic surgery, in line with NLST and NELSON trial criteria. Others state that screening should not include those with <10-year life expectancy (American College of Chest Physicians),^40^ those with life-limiting comorbid conditions (American Cancer Society),^41^ or as long as patient functional status and comorbidity allow consideration for curative intent therapy (National Comprehensive Cancer Network).^42^ This array of recommendations creates confusion for clinicians and has fallen short of its goal. Defining who is and is not healthy enough for screening may be particularly important for LCS because the criteria used to identify individuals eligible for screening (advanced age and heavy smoking history) also increase the risk of dying for a variety of other reasons.

There is an urgent need for more research in this area to help patients and clinicians rationally weigh the risks and benefits of LCS in the context of an individual's health status,^11^ as the first step in shared decision making, a fundamental component of LCS^43^^,^^44^ that is underutilized.^45^^,^^46^ If LCS moves toward risk-based eligibility, then consideration of overall health and life expectancy will be even more important because such strategies tend to select older individuals who may be frailer.^12^

The racial and ethnic differences in LCS use among veterans merit urgent attention. Previous work by our group^21^ corroborates a growing body of literature that racial and ethnic disparities exist in many aspects of LCS.^47^^,^^48^ These must be understood and addressed urgently to promote equity^48^ and because LCS may be particularly beneficial in Black individuals.^16^ Updated 2021 LCS guidelines may help to increase the use of LCS among Black individuals on the basis of early cross-sectional research.^10^ Nonetheless, the very low prevalence of LCS among non-White veterans (6.2%) should prompt further investment of resources to understand the barriers to LCS access for this group because this has the potential to magnify existing disparities in lung cancer outcomes.^49^

Strengths of this study include the population-based survey design, which allows for population-level inferences that are likely generalizable to the veteran populations of the 28 states that included the optional LCS module. We chose a measure of health status combining self-rated health and the ability to climb stairs that is highly relevant to LCS and could be replicated in future research. Self-rated health and ability to climb stairs provide a clear point of comparison with randomized trials; NELSON investigators excluded those who rated their health as moderate/bad and could not climb 2 flights of stairs.^4^ Furthermore, stair climbing is a common preoperative measure of cardiovascular fitness,^50^ and self-rated health predicts all-cause mortality more accurately than a 30-item objective measure.^51^^,^^52^

### Limitations

Similar to other analyses using BRFSS data to study LCS, this study has several limitations, including the self-reported nature of variables, including smoking history, LCS, race/ethnicity, health status, and veteran status. However, we have no reason to suspect that the accuracy of self-reported variables would vary differentially across categories defined by veteran status, health status, or race/ethnicity; thus, any misclassification would bias our estimates toward the null. In addition, BRFSS has a limited proportion of non-White individuals who responded to the survey (9.6% in this analysis, unweighted). Owing to low numbers of individuals in subcategories defined by race and ethnicity, our analysis relies on aggregated results (White/non-White), which may obscure important differences between groups of individuals who identify as non-White.^28^ We were unable to directly ascertain mortality risk, although we relied on self-reported health, which is validated to predict mortality,^51^ and ability to climb stairs, which is a common measure of preoperative fitness.^50^ Furthermore, these metrics are directly relevant to LCS given their use as exclusion criteria in NELSON.^4^ The cross-sectional nature of the study design precludes temporal sequencing of exposure and outcome. Similar to other BRFSS analyses of LCS, we were unable to exclude diagnostic imaging studies ordered in response to signs/symptoms of lung cancer or other pathology, which must be addressed in future research on the use and outcomes of LCS to obtain unbiased results. The BRFSS survey question assessing LCS use does not differentiate between initial LDCT and subsequent scans, and different steps along the process of LCS may be associated with different barriers for patients.^20^^,^^53^ Furthermore, the answer choices for self-rated health in the BRFSS (excellent/very good/good/fair/poor) differ somewhat from those used by NELSON investigators because they excluded individuals with moderate or bad self-rated health, and the question that ascertained difficulty in climbing stairs also asked about difficulty in walking in general.

Although we could not restrict to veterans receiving care through the VHA, these results may still be useful to VHA, which has embarked on a multimillion dollar endeavor^9^ to meet the large unmet need for LCS among veterans.^54^ The VHA may be uniquely poised to deliver high-quality LCS, as the largest integrated health system in the U.S. and one with an established track record of implementing high-quality cancer screening.^17^^,^^55^ This analysis suggests unique opportunities for implementation of LCS in the VHA. First, the VHA's electronic medical record already includes automated information to ascertain life expectancy,^56^ which is a key consideration for LCS and could improve the identification and utilization of LCS among healthier veterans. Second, the military's integrated healthcare model has a long track record of reducing racial disparities, including in cancer care.^57^ The racial and ethnic disparities observed in this study may reflect broader disparities in the U.S. healthcare system^58^ because of structural racism,^59^ which the VHA is uniquely positioned to address given its integrated care model.

## CONCLUSIONS

LCS use is strongly correlated with poorer health among veterans, and one third of screened veterans would not qualify for a pivotal trial of LCS on the basis of poor health. It is unclear whether actively screening for asymptomatic lung cancer provides more benefit than harm for these veterans or whether they may be better served by focusing on their other, known health issues. Marked screening disparities exist between White and non-White veterans, despite likely greater screening mortality benefits among Black individuals. These results add to the importance of current efforts to increase LCS and support additional outreach to screen healthier veterans, especially non-White veterans who are appropriate candidates for screening.

## Appendix Supplementary materials

Image, application 1

Image, application 2

Image, application 3

Image, application 4

## Footnotes

Supplementary material associated with this article can be found in the online version at doi:10.1016/j.focus.2023.100084.
