# Supplementary material for: Lung Cancer Screening Among U.S. Military Veterans by Health Status and Race and Ethnicity, 2017–2020: A Cross-Sectional Population-Based Study
Source: AJPM Focus. 2023 Feb 9;2(2):100084. doi: 10.1016/j.focus.2023.100084 (PMC10546514; doi:10.1016/j.focus.2023.100084)
Supplement: Supplementary file 1 [file mmc1.docx]

**Appendix Table 1. LCS (A) eligibility and (B) prevalence among veterans and non-veterans under 2013 guidelines, 2017-2020.**

| (A) LCS eligibility | | | |
| --- | --- | --- | --- |
|  | Total potentially eligible for LCS^a^  (unweighted n) | Eligible for LCS  (unweighted n) | LCS-eligible (weighted proportion, 95% CI) |
| Non-veterans | 117,451 | 11,377 | **10.2 (9.7-10.7)** |
| Veterans | 21,504 | 3,435 | **17.0 (15.7-18.2)***** |
| (B) LCS prevalence | | | |
|  | Total eligible for LCS^b^  (unweighted n) | Received LCS  (unweighted n) | Received LCS (weighted proportion, 95% CI) |
| LCS-eligible non-veterans | 11,160 | 1,674 | 16.3 (14.0-18.5) |
| LCS-eligible veterans | 3,376 | 573 | 19.4 (15.8-22.9) |

^a^ Total number of survey respondents in 28 states utilizing the optional LCS module, who were 55-79 years old, with no personal history of lung cancer, and with complete age and smoking history to calculate lung cancer screening eligibility.

^b^ Excludes n=276 with unknown screening status (<2% of total eligible).

Boldface indicates statistical significance (****p*<0.001).
