# Supplementary material for: Lung Cancer Screening Among U.S. Military Veterans by Health Status and Race and Ethnicity, 2017–2020: A Cross-Sectional Population-Based Study
Source: AJPM Focus. 2023 Feb 9;2(2):100084. doi: 10.1016/j.focus.2023.100084 (PMC10546514; doi:10.1016/j.focus.2023.100084)
Supplement: Supplementary file 2 [file mmc2.docx]

**Appendix Table 2. Detailed race and ethnicity among veterans eligible for lung cancer screening in 28 states, 2017-2020.**

| Race and ethnicity^a^ | Veterans (unweighted n=3,376),  weighted % (95% CI) |
| --- | --- |
| Non-Hispanic White | 86.3 (82.5-89.4) |
| Non-Hispanic Black | 9.1 (6.5-12.6) |
| American Indian or Alaska Native | 1.2 (0.8-2.0) |
| Asian | 0.6 (0.09-4.2) |
| Native Hawaiian or other Pacific Islander | 0.05 (0.007-3.2) |
| Other | 0.3 (0.2-0.6) |
| Multiracial | 1.3 (0.9-2.0) |
| Hispanic | 1.0 (0.6­-1.7) |

^a^ Race and ethnicity were self-reported by respondents, within these pre-defined survey categories.
