# Supplementary material for: Lung Cancer Screening Among U.S. Military Veterans by Health Status and Race and Ethnicity, 2017–2020: A Cross-Sectional Population-Based Study
Source: AJPM Focus. 2023 Feb 9;2(2):100084. doi: 10.1016/j.focus.2023.100084 (PMC10546514; doi:10.1016/j.focus.2023.100084)
Supplement: Supplementary file 3 [file mmc3.docx]

**Appendix Table 3. Use of LCS among veterans by detailed race and ethnicity, 2017-2020.**

| Race and ethnicity | Did not receive LCS (unweighted n) | Received LCS (unweighted n) | Proportion received LCS  (weighted %, 95% CI) | Unadjusted RR (95% CI) | Adjusted RR  (95% CI)^a^ |
| --- | --- | --- | --- | --- | --- |
| Non-Hispanic White | 2466 | 521 | 21.7 (17.7-25.7) | 1.00 (referent) | 1.00 (referent) |
| Non-Hispanic Black | 99 | 13 | 6.2 (1.1-11.2) | 0.28 (0.12-0.66) | 0.36 (0.13-0.91) |
| American Indian or Alaska Native | 61 | 10 | 4.7 (0.1-9.3) | 0.22 (0.08-0.58) | 0.12 (0.03-0.44) |
| Asian | 2 | 1 | 1.6 (-2.8-6.0) | 0.07 (0.00-0.96) | 0.97 (0.11-3.42) |
| Native Hawaiian or other Pacific Islander | 2 | 0 | 0.0 (omitted) | 1.00 (empty) | 1.00 (empty) |
| Other | 28 | 4 | 2.0 (-0.3-4.3) | 0.09 (0.03-0.30) | 0.08 (0.02-0.34) |
| Multiracial | 64 | 9 | 5.9 (-0.9-12.7) | 0.27 (0.08-0.83) | 0.35 (0.09-1.14) |
| Hispanic | 31 | 4 | 14.1 (-3.8-32.0) | 0.65 (0.16-1.95) | 0.58 (0.14-1.81) |
| Missing | 50 | 11 |  |  |  |

Abbreviations: CI=confidence interval. RR=relative risk.

Race and ethnicity were self-reported by respondents, within these pre-defined categories in the survey.

^a^ Adjusted for race (white/non-white), age (5 year categories), sex (male/female), marital status (married/divorced/widowed/separated/never married/part of an unmarried couple), BMI (<18.5 kg/m2, 18.5-<25 kg/m2, 25-<30 kg/m2, 30+ kg/m2), education (never attended school or only kindergarten/elementary or middle school/some high school/high school graduate/some college/college graduate or more), smoking history in pack-years (quartiles), health insurance status (any/none), receipt of influenza vaccine in prior 12 months (no/yes), difficulty paying for medical care (no/yes), diagnosis of chronic obstructive pulmonary disease (no/yes), personal history of non-lung cancer (no/yes), and survey year (2017/2018/2019/2020).
