# Supplementary figures and images for: Lung Cancer Screening Among U.S. Military Veterans by Health Status and Race and Ethnicity, 2017–2020: A Cross-Sectional Population-Based Study
Source: AJPM Focus. 2023 Feb 9;2(2):100084. doi: 10.1016/j.focus.2023.100084 (PMC10546514; doi:10.1016/j.focus.2023.100084)

**Appendix Figure**. Study flowchart.


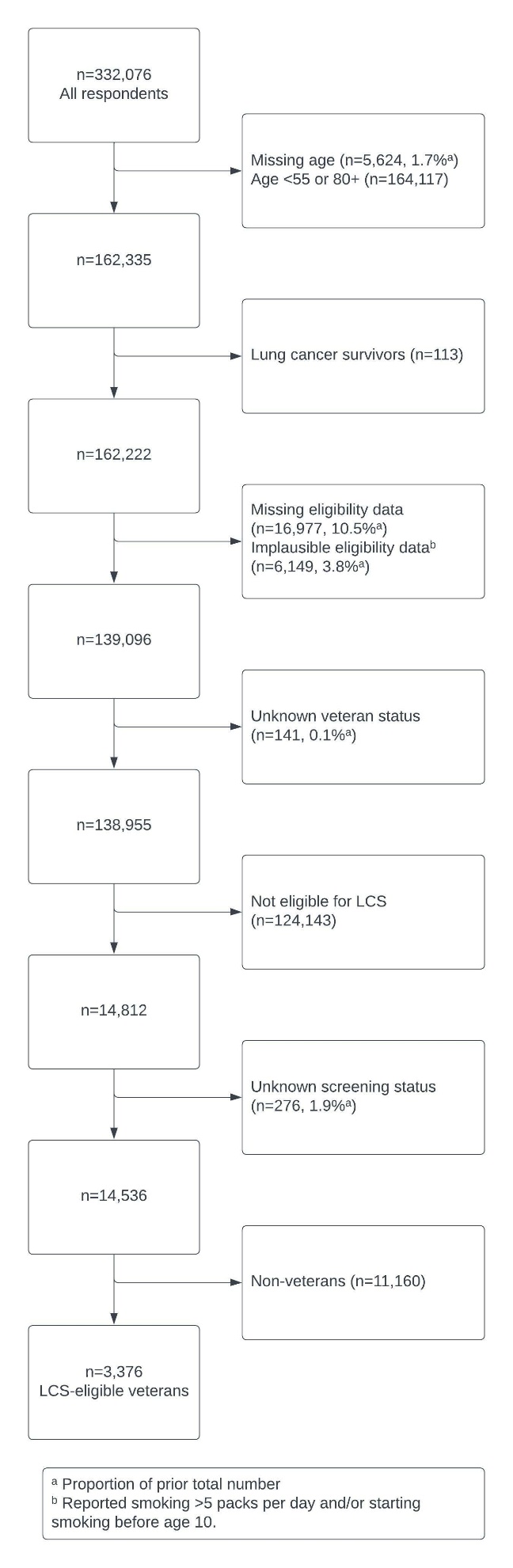

Supplement: Supplementary file 4 [file mmc4.docx]
